# Transcriptional Reprogramming by AP-1-Bound Cis-Regulatory Elements Is Associated with Melanoma Development

**DOI:** 10.3390/ijms27146459

**Published:** 2026-07-21

**Authors:** Zubeir El Ahmad, Katrin Ludwig, Alexander O. Matthies, Anja Katrin Bosserhoff, Melanie Kappelmann-Fenzl

**Affiliations:** 1 Institute of Biochemistry, Friedrich-Alexander-Universität Erlangen-Nürnberg (FAU), 91054 Erlangen, Germany; zubeir.el-ahmad@th-deg.de (Z.E.A.); katrin.kl.ludwig@fau.de (K.L.); alexander.matthies@fau.de (A.O.M.); 2 Faculty of Computer Science, Deggendorf Institute of Technology, 94469 Deggendorf, Germany

**Keywords:** malignant melanoma, AP-1 transcription factors, epigenetic regulation, enhancer-associated transcriptional regulation

## Abstract

Malignant melanoma is characterized by high metastatic potential and cellular plasticity. Its progression is driven not only by genetic alterations but also by epigenetic reprogramming and changes in the transcriptome, mediated by transcription factors. The AP-1 family favors an invasive cell state by facilitating processes such as angiogenesis and migration. However, the AP-1-driven regulatory landscape and its implications for melanoma plasticity remain poorly understood. Here, we further elucidate the role of the AP-1 members c-Jun and Fra-1 in enhancer-related gene regulation during melanoma development. Integrative ChIP-seq and RNA-seq analyses revealed extensive enhancer reprogramming during tumor formation and significant upregulation of genes annotated to enhancers gained in melanoma cells. On average, half of these regions are directly bound by c-Jun/Fra-1, given a high AP-1 motif rate (~80%) and chromatin accessibility. Genes associated with c-Jun/Fra-1-bound enhancer regions are functionally linked to an invasive phenotype and comprise well-known melanoma drivers involved in migration and EMT. Clinically, high c-Jun/Fra-1 and target gene expression correlate with poor survival in *BRAF* wild-type and *NRAS* mutant melanoma patients, highlighting c-Jun/Fra-1 as a potential biomarker and therapeutic target. Overall, our data identify c-Jun/Fra-1 as important contributors to melanoma development through enhancer-mediated transcriptional programs.

## 1. Introduction

Malignant melanoma is an aggressive skin cancer originating from melanocytes. Although it accounts for only ~1% of skin cancers, it is responsible for the majority of skin cancer-related deaths due to its high metastatic potential and therapy resistance [1,2]. Mortality is affected by late diagnosis and the tumor’s ability to adapt and evade immune surveillance. Recent therapeutic advances, including immune checkpoint inhibitors and BRAF/MEK-targeted therapies, have improved outcomes, but resistance and relapse remain major challenges [3,4]. Key oncogenic drivers include BRAF, NRAS, and NF1, which activate MAPK and PI3K–AKT pathways. Additional susceptibility genes include *CDKN2A*, *CDK4*, *TERT*, and *MC1R*, as well as emerging new candidates [2,5].

Beyond genetic mutations, melanoma development and progression are strongly influenced by transcriptional deregulation. A key step is the deregulation of cancer-supporting transcription factors, particularly the activating protein-1 (AP-1) family, which includes JUN, FOS, ATF, and MAF subfamilies. AP-1 proteins bind to the consensus sequence 5′-TGA(C/G)TCA-3′ to regulate gene expression and influence cancer-related signaling pathways. The AP-1 member c-Jun acts as a central regulator of proliferation and invasion, particularly in *PTEN* wild-type melanoma cells, where its activity drives aggressive phenotypes and supports survival signaling. Genome-wide analyses have identified direct c-Jun target genes involved in cell cycle control, migration, and invasiveness, underscoring its role in metastatic competence [6]. c-Jun stability and nuclear translocation depend on complex formation with α-tubulin and importin 13, highlighting a mechanistic layer that ensures sustained transcriptional activity [7]. Furthermore, microRNA-mediated regulation, such as the loss of miR-125b in melanoma, also targeting c-Jun, demonstrates how post-transcriptional control influences melanoma progression [8]. Fra-1 (*FOSL1*), another AP-1 (FOS) family member, has emerged as an important regulator of melanoma progression by controlling gene expression programs linked to tumor growth and invasion. Recent work demonstrates that Fra-1 mediates oncogenic transcriptional networks and contributes to melanoma metastasis [9]. Mechanistically, Fra-1 has been shown to regulate AP-1-dependent gene expression and cooperate with signaling pathways such as MAPK to drive malignant behavior [10]. More recent studies further highlight its role in shaping melanoma cell states and transcriptional plasticity, reinforcing *FOSL1* as a key functional contributor to melanoma progression [11]. Together, these factors contribute to melanoma plasticity and therapy resistance, positioning c-Jun/Fra-1 dimers as a critical node for targeted intervention. The transcriptionally active AP-1 dimers are highly heterogeneous because multiple subunits can be expressed simultaneously. While studies show that dimer composition affects binding site selection, the target genes of defined AP-1 dimers driving melanomagenesis remain unclear [6,12,13]. A balanced AP-1 network governs cellular plasticity, and its perturbation predictably alters cellular heterogeneity, highlighting AP-1 as a potential therapeutic target [14].

In addition, epigenetic mechanisms such as DNA methylation, histone modifications, and chromatin remodeling are fundamental to melanoma development and progression [15,16]. Specific histone marks are known to define different regulatory states: H3K4me3 is enriched at active promoters, H3K27ac marks active enhancers and promoters, and H3K4me1 identifies poised or active enhancers [17,18]. Enhancers exert their influence on transcriptional reprogramming by serving as dynamic regulatory hubs that integrate chromatin architecture and transcription factor activity to control gene expression. These elements recruit transcription factors and cofactors to establish active chromatin states and facilitate long-range interactions with target promoters [17,18]. Mechanistically, this process involves remodeling of nucleosome positioning, changes in histone acetylation, and cooperative binding of transcription factors such as AP-1, which collectively shift transcriptional networks toward tumor-promoting states [14,19]. Recent studies emphasize the interplay between genetic and epigenetic changes, tumor microenvironment, and metabolic reprogramming in melanoma development and progression. Understanding these mechanisms is critical for developing next-generation therapies targeting transcriptional dependencies and overcoming resistance to current treatments [2,20].

In this study, we demonstrate that the epigenetic landscape underlies significant rearrangements during malignant transformation and that AP-1 members c-Jun and Fra-1 play an essential role in the distal regulation of tumor-promoting target genes by binding to melanoma-gained enhancers, having a major impact on melanoma development.

## 2. Results

### 2.1. Identification of Melanoma-Associated Enhancer Regions

**Figure 1:**
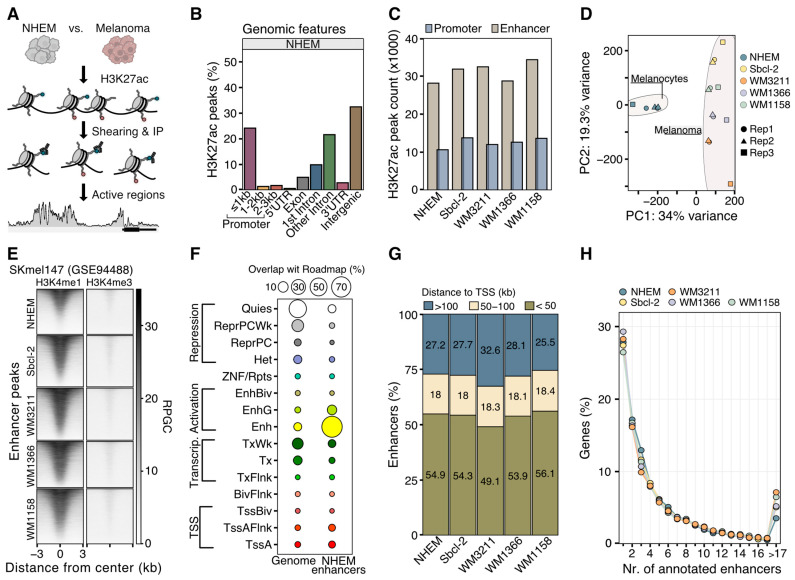
Defining distal cis-regulatory regions in NHEMs and melanoma. (**A**) Experimental setup of ChIP-seq experiments using normal human epidermal melanocytes (NHEMs) and 4 different melanoma cell lines (Sbcl-2, WM3211, WM1366, WM1158) with an H3K27ac-specific antibody to precipitate transcriptionally active regions. (**B**) Distribution of NHEM-derived H3K27ac ChIP-seq peaks in annotated genomic features. (**C**) H3K27ac ChIP-seq peak counts in NHEMs and melanoma cells annotated to promoters or enhancers. Assignment according to the distance to the nearest TSS (threshold: 3kb). (**D**) Principal Component Analysis of NHEMs and melanoma cell line H3K27ac ChIP-seq samples based on potential enhancers. Replicates are indicated by shape. (**E**) Histogram showing the H3K4me1 and H3K4me3 ChIP-seq signal of publicly available data from the melanoma cell line (SKmel147) (GSE94488) around the defined enhancer peaks in NHEMs, Sbcl-2, WM3211, WM1366, and WM1158. (**F**) Roadmap core 15-state chromatin state of human foreskin melanocytes over the total genome compared to chromatin states at potential enhancers identified in NHEMs. (**G**) Distribution of enhancers identified in NHEMs, Sbcl-2, WM3211, WM1366, and WM1158 by their distance to the nearest assigned TSS (>100 kb, 50–100 kb, <50 kb). (**H**) Proportion of genes assigned to the number of potential enhancers.

Epigenetic changes play a crucial role in the regulation of gene expression and thus during transcriptional reprogramming towards malignancy. The importance of the epigenetic landscape in transcriptional regulation of tumor-relevant genes is well known, but only poorly understood so far. This study aims to elucidate epigenetically modified regulatory features of the DNA with an impact on melanoma-associated gene expression affecting cellular behavior. Therefore, we performed ChIP-seq experiments using 4 different melanoma cell lines (Sbcl-2, WM3211, WM1366, WM1158) and normal human epidermal melanocytes (NHEMs) with an H3K27ac-specific antibody to precipitate transcriptionally active regions (Figure 1A and Supplementary Table S1). Correlation-based clustering revealed the best grouping by condition replicates (Supplementary Figure S1A) and indicated differences between NHEMs and melanoma cell lines, as well as among melanoma cell lines. These results already suggested the relevance of epigenetic changes during melanoma development and progression. Examining the genomic distribution of identified H3K27ac ChIP-seq peaks surprisingly showed that most peaks occurred in intergenic or intronic regions and fewer peaks in annotated promoter regions located relatively close to the respective TSS (≤1 kb) (Figure 1B, Supplementary Figure S1B). Thus, we speculated whether those non-promoter annotated H3K27ac ChIP-seq signals could be located in proximal or distant cis-regulatory elements. The initial assignment of the H3K27ac peaks to potential enhancers or promoters was carried out according to their distance to the nearest TSS, with a threshold of 3kb, resulting in a comparable number of potential regulatory regions from NHEMs and melanoma cell lines and an overall ratio of promoters to enhancers of approximately 1:3 (Figure 1C).

Furthermore, a Principal Component Analysis (PCA) including the identified H3K27ac status from potential enhancer elements of the different cell lines showed a distinct separation between melanocytes and melanoma cells, suggesting changes in the epigenome during transformation (Figure 1D). Our findings were further verified by the inclusion of publicly available ChIP-seq data from another melanoma cell line (SKmel147) and melanocytes (NHM), respectively (GSE94488) [21]. Here, a strong H3K4me1 enrichment was detected in all melanoma cell lines as well as in NHEMs within cis-regulatory regions classified as potential enhancers based on the H3K27ac signal, compared to an almost absent H3K4me3 signal (Figure 1E). Conversely, the potential promoter regions (H3K27ac signature 3kb around the nearest TSS) showed nearly no H3K4me1 signal, whereas the H3K4me3 signature matches the H3K27 acetylated genomic regions annotated to promoters (Supplementary Figure S1C). In addition, the enhancer-defining H3K4me1 signal in SKmel147 and NHM for distal cis-regulatory regions identified in the melanoma cell lines studied behaves inversely to each other. NHEMs match the H3K4me1 signature of NHM, but not the melanoma cell lines, and vice versa (Supplementary Figure S1D). This led us to the assumption that the respective detected H3K27ac ChIP-seq peaks annotated to intergenic or intronic genomic regions, together with the supporting H3K4me1 signature, are most likely associated with active enhancer elements [17,18].

To confirm that the identified cis-regulatory elements are enhancers, we utilized additional public resources focusing on epigenetic landscapes. A comparison with the Roadmap database demonstrated that roughly 90% of potential enhancers identified in NHEMs actually align with active enhancer-defined chromatin (Figure 1F; Enh, EnhG) of the Core 15-state chromatin model derived from human foreskin melanocytes, representing a substantial increase relative to the total genome, comprising only about 12% active enhancer chromatin. Additionally, overlaying ENCODE enhancer (ELS) and promoter (PLS) annotations from all available bio samples (no specific melanoma or melanocyte samples available) with our newly identified potential enhancer elements revealed that ~95% match with ELS, while this applies for only 5% of PLS signatures (Supplementary Figure S1E). In accordance with typical distances of previously described and annotated enhancers [22], around 2/3 of the potential enhancer elements are located ≤100kb to the nearest assigned TSS (Figure 1G). Interestingly, assigning genes to these potential enhancer regions resulted in ~60% of the genes being annotated to 3 or fewer potential enhancers and can thus be attributed to a rather specific enhancer association (Figure 1H).

Taken together, our data, in accordance with published studies, clearly reveal that the detected distal H3K27ac signature in melanoma cell lines and NHEMs can be defined as cis-regulatory elements (enhancers), which might play an important role in epigenetic reprogramming during melanoma development and thus gene expression regulation affecting the cellular phenotype.

### 2.2. Melanoma Development Is Accompanied by Reprogramming of the Enhancer Landscape

**Figure 2:**
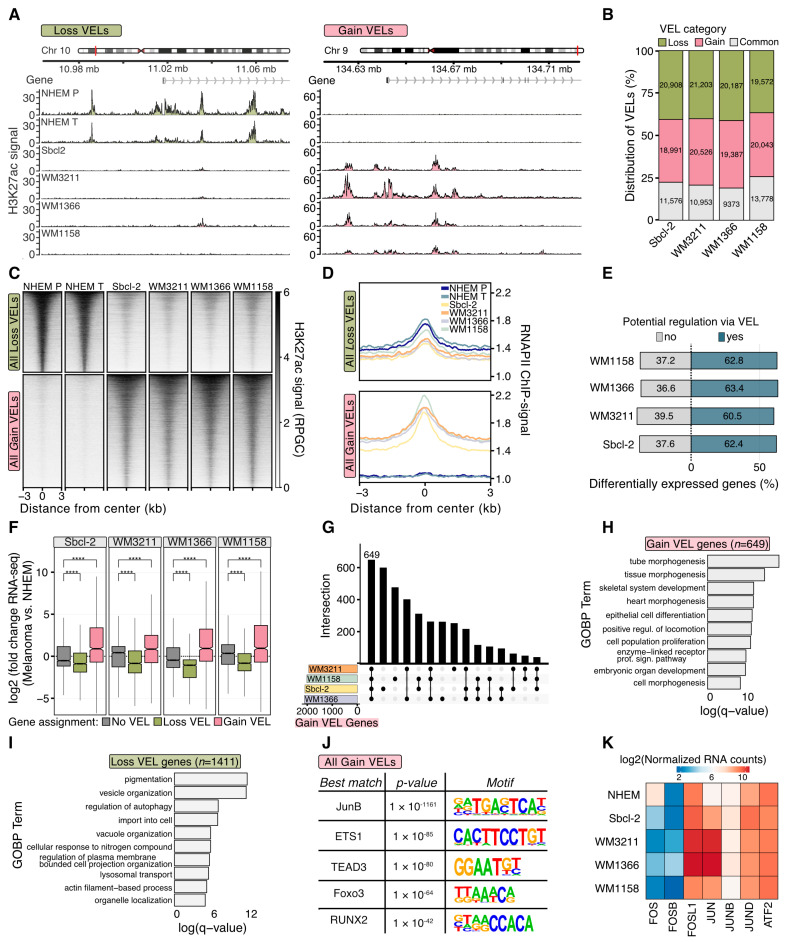
Characteristic features of differentially enriched enhancers (**A**) IGV illustrations exemplarily depicting the H3K27ac ChIP-seq signals at Loss and Gain Variant Enhancer Loci (VELs) in NHEMs and melanoma cell lines. (**B**) Distribution of Loss VELs, Gain VELs, and Common VELs in the melanoma cell lines. Absolute values for each category are indicated. Differential enrichment analysis was performed on an individual consensus peak set for each cell line, generated by merging NHEM enhancers with melanoma-derived enhancers from the respective cell line. Three biological H3K27ac ChIP-seq replicates were included for every cell line used. Counts were normalized using DESeq2’s median-of-ratios method, and dispersion estimates were obtained using DESeq2’s parametric mean-dispersion model with empirical Bayes shrinkage. (**C**) Histogram showing the H3K27ac ChIP-seq signal around the enhancer center of all Loss and Gain VELs in NHEMs (NHEM_P, NHEM_T) and melanoma cell lines. (**D**) RNAPII ChIP-seq signal intensity around the enhancer center in all Loss and Gain VELs in NHEMs (NHEM_P, NHEM_T) and melanoma cell lines. The signals for the two biological RNAPII ChIP-seq samples per cell line were merged. (**E**) Proportion of DEGs per melanoma cell line potentially regulated or not regulated by an identified Gain or Loss VEL. (**F**) Log2 fold-change RNA-seq expression values of genes assigned to No VEL, Loss VEL, and Gain VEL. (**** *p* < 0.0001, Wilcoxon test, unpaired, two-sided). (**G**) Intersection of annotated target genes to Gain VELs in melanoma cell lines. Number of genes found in all cell lines is indicated. Total gene numbers are shown in horizontal bar graphs. (**H**) Functional gene set enrichment analysis of Gain VEL-associated DEGs upregulated in all melanoma cell lines (*n* = 649). (**I**) Functional gene set enrichment analysis of Loss VEL-associated DEGs downregulated in all melanoma cell lines (*n* = 1411). Statistical significance for the GO enrichment analyses was calculated using a hypergeometric test, and multiple-testing correction was performed using the Benjamini–Hochberg method. (**J**) Motifs derived from de novo motif analysis enriched in all Gain VELs. *p*-values and transcription factors showing the best match with the de novo motifs are indicated. (**K**) Expression values of AP-1 family members given as log2 normalized RNA-seq counts.

Next, to focus on cancer-relevant differences in the epigenetic landscape, we aimed to identify cis-regulatory regions specific to melanoma and melanocytes using differential enrichment analysis. This approach allowed us to classify the regions into three variant enhancer loci (VEL) groups: those lost during the transition of melanocytes to melanoma (Loss), those active in both stages (Common), and regions newly gained in melanoma (Gain) (Figure 2A). Overall, only ~20% of enhancers fall into the Common VEL group, while the largest proportion appears to be specific to either melanocytes or melanoma cells, with an equal distribution between the two categories (Figure 2B and Supplementary Table S2). Hence, compared to the H3K27ac signature of NHEMs, the melanoma cell lines indicate an epigenetic reprogramming either by Loss or Gain VELs. Furthermore, although a few cell line-specific VELs were identified, the H3K27ac signal intensity generally follows a consistent trend in both the Gain and Loss categories. In NHEMs, we observed a high acetylation rate in all Loss VELs compared to all Gain VELs, while the melanoma cells illustrated a contrasting behavior in this regard (Figure 2C). This was also confirmed by applying the public H3K4me1 ChIP-seq data from NHM and SKmel147 to the Gain and Loss VELs identified in this study. Here, a strong accumulation of H3K4 mono-methylation was observed in NHM at regions of the detected Loss VELs with a corresponding signal decrease in SKmel17, whereas an inverse methylation pattern was recognizable for the Gain VELs (Supplementary Figure S2A). Since it is well known that short enhancer RNAs (eRNA) are transcribed at active enhancers by RNA Polymerase II (RNAPII) [17], we performed ChIP-seq experiments using an RNAPII antibody as an additional layer of proofing transcriptional activity at the newly identified enhancers. In accordance with the assumption, a marked increase in ChIP-seq signal intensity and thus RNAPII occupancy was evident in Gain VELs concerning the melanoma cells, as well as in Loss VELs regarding the NHEMs, since these regions are only active in melanocytes (Figure 2D). To avoid that these signal patterns mainly derive from actively gene-transcribing RNAPII, we reevaluated the occupancy while excluding intragenic VELs (Supplementary Figure S2B). However, the comparison between intergenic-only and all VELs revealed a high degree of similarity, confirming that the observed effects are not driven by RNAPII occupancy associated with ongoing gene transcription within gene bodies.

Further, we were interested in the influence of the observed epigenetic differences comparing NHEMs and melanoma cells on gene expression and hence integrated bulk RNA-seq data of the respective cell lines in our analysis (GSE284779). On a global scale, analyzing transcriptomic alterations driven by enhancer changes revealed that approximately 62% of differentially expressed genes (DEGs, Supplementary Table S3) in each melanoma cell line were annotated to a Gain or Loss VEL (Figure 2E). Moreover, in line with the H3K27ac ChIP-seq, RNA-seq integration yielded a positive correlation between changes in gene expression and assignment of the corresponding VEL group (Common, Loss, Gain). More precisely, DEGs associated with a Gain VEL showed significantly increased expression (log2FC > 0) compared to differential genes with no VEL assigned, and those DEGs with an associated Loss VEL were significantly downregulated (Figure 2F, **** *p* < 0.0001, Wilcoxon test). This positive correlation is also evident for the number of VELs assigned per gene and the magnitude of fold change (Supplementary Figure S2C).

Interestingly, the largest proportion of genes was consistently regulated in all melanoma cell lines, both for the Gain (~1/3) and Loss (~1/2) group, which became evident by intersecting the annotated target genes (Figure 2G, Supplementary Figure S2D). From a functional perspective, the Gain VEL-associated DEGs commonly upregulated in all melanoma cell lines (*n* = 649) were connected to melanoma-related traits such as morphogenesis and locomotion, while the overlap of Loss VEL genes (*n* = 1411) was enriched in GOBP terms associated with melanocyte-specific behavior (Figure 2H,I). To better understand the molecular mechanisms underlying the enhancer reprogramming in melanoma, we aimed at determining key transcription factors involved in VEL-mediated transcriptional changes.

Applying HOMER’s de novo motif analysis identified the AP-1 motif as the best match for all Gain VELs, followed by ETS and TEAD (Figure 2J). In the Loss group, significant enrichment was found for the MITF and SOX10 motifs (Supplementary Figure S2E). These findings were confirmed by overlaying known transcription factor binding sites, obtained from public ChIP-seq data across different tissues, onto the Gain and Loss VEL regions using the CistromeDB toolkit. Here too, SOX10 and MITF showed a high overlap with Loss VELs, whereas AP-1 was present as the most prominent transcription factor group for Gain VELs (Supplementary Figure S2F,G). To further assess regulatory network activity underlying melanoma-associated H3K27ac remodeling, we performed CREMA analysis on the H3K27ac ChIP-seq data aggregated into NHEM and melanoma groups. This analysis inferred increased melanoma-associated activity of several AP-1-family or AP-1-associated regulatory matrices, thereby independently supporting the relevance of AP-1 signaling and our focus on Fra-1/c-Jun (Supplementary Figure S2H,I). To pinpoint AP-1 family members of particular importance, we analyzed their expression status in our RNA-seq data and determined high expression levels for Fra-1 (*FOSL1*) and c-Jun (JUN) in all examined melanoma cells (Figure 2K). Moreover, analyzing the co-expression status of selected members revealed the strongest positive expression correlation between Fra-1 and c-Jun (Spearman correlation: 0.65; Supplementary Figure S2J). Since the AP-1 family members Fra-1 and c-Jun are well known to play a crucial role in the development and progression of melanoma, we concentrated on these transcription factors and their contribution to enhancer-linked gene expression regulation.

### 2.3. AP-1 Binding Marks Melanoma-Gained Distal Regulatory Elements Linked to Gene-Expression Changes

**Figure 3:**
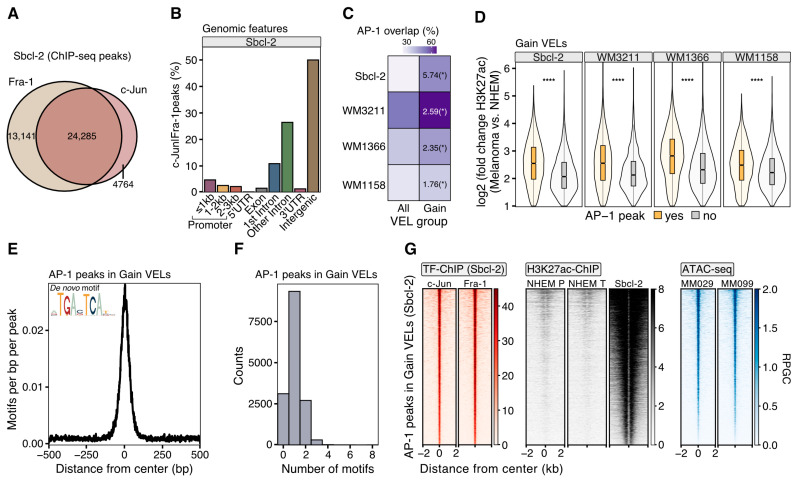
Overlap between c-Jun/Fra-1 binding sites and Gain VELs (**A**) Venn Diagram showing the overlap between c-Jun and Fra-1 binding sites in Sbcl2 (PRJNA1197724). (**B**) Distribution of Sbcl-2 derived c-Jun| Fra-1 ChIP-seq peaks in annotated genomic features. (**C**) Proportion of c-Jun/Fra-1 overlap with all VELs and Gain VELs only. Relative proportion is color-coded. Fisher’s exact test compares the overlap of c-Jun/Fra-1 in Gain VELs with All VELs for each melanoma cell line. Odd scores are indicated. (* *p* < 0.05). (**D**) Log2 fold-change H3K27ac ChIP-seq signatures of Gain VEL with and without overlapping c-Jun/Fra-1 binding sites. (**** *p* < 0.0001, Wilcoxon test, unpaired, two-sided). (**E**) Histogram showing the distribution of the most significant de novo-derived motif within all c-Jun/Fra-1 peaks overlapping Gain VELs. (**F**) Number of the de novo-derived AP-1 motif per c-Jun/Fra-1 peaks overlapping Gain VELs. (**G**) Histograms showing the signals of c-Jun/Fra-1 ChIP-seq, H3K27ac ChIP-seq, and ATAC-seq (GSE134432) at c-Jun/Fra-1 binding sites overlapping Gain VELs in Sbcl-2.

Based on our findings, we further focused on elucidating the involvement of AP-1 family members c-Jun and Fra-1 in enhancer-related gene expression regulation. Therefore, c-Jun- and Fra-1 ChIP-seq data of Sbcl2, WM3211, WM1366, and WM1158 were utilized (PRJNA1197724). Transcription factor peak calling resulted in a high number of detected binding locations for both factors in each melanoma cell line, with a high degree of overlapping sites and an even genomic distribution between c-Jun and Fra-1 (Supplementary Figure S3A). However, we only considered binding regions where a significant peak was called for both c-Jun and Fra-1 to focus on biologically meaningful sites (Figure 3A, Supplementary Figure S3B). As it is known that c-Jun and Fra-1 can form heterodimers that play a pivotal role in the regulation of gene expression [23], we were further interested in their contribution to acquiring a malignant phenotype. Surprisingly, genomic annotation of the overlapping c-Jun/Fra-1 peaks revealed most binding sites (~90%) being located in intergenic as well as intron regions (Figure 3B, Supplementary Figure S3C), similar to our H3K27ac ChIP-peaks (Figure 1B, Supplementary Figure S1B), but much more dominant. When integrating c-Jun/Fra-1 ChIP-seq data with our enhancer regions, the vast majority of AP-1 peaks overlapping with VELs appear to coincide with one of the two transcriptionally active categories in melanoma, Common or Gain, with the latter one accounting for the largest share at approximately 60% (Supplementary Figure S3D, pink bar). In contrast, AP-1 binding was detected in only a small fraction (~10%) of Loss VELs in melanoma cells (Supplementary Figure S3D, green bar). Furthermore, binding of c-Jun and Fra-1 was significantly overrepresented within Gain VELs compared to the total set of all VELs detected, with overlaps between c-Jun/Fra-1 and Gain VELs ranging from roughly 30% to 70% (odd-scores; Figure 3C). Integrating H3K27ac signatures (log2FC) demonstrated a significant association between the acetylation signal intensity and c-Jun/Fra-1 binding in Gain VELs (Figure 3D), emphasizing the close connection between AP-1 occupancy, enhancer-associated chromatin activity, and melanoma-gained distal regulatory elements.

The DNA-binding mechanism of AP-1 transcription factors, including the c-Jun/Fra-1 heterodimer, is well characterized, particularly their recognition of the consensus sequence 5′-TGA(C/G)TCA-3′ [24]. However, AP-1 binding modalities in distal cis-regulatory elements are poorly investigated so far, and we speculated whether this might be a promoter-like case, where gene expression regulation occurs via direct DNA-binding. Indeed, a subsequent motif analysis clearly supported our assumption, as ~80% of the screened c-Jun/Fra-1 binding sites in Gain VELs display a central consensus AP-1 motif, with mostly a single motif occurring per ChIP-seq peak, indicating specific binding of one dimer (Figure 3E,F). Nevertheless, transcription factor binding and regulation require not only motif occurrence but also an appropriate chromatin conformation. Thus, we reviewed the H3K27 acetylation around the c-Jun/Fra-1 binding sites in Gain VELs, yielding a bimodal distribution pattern known to be characteristic of transcription factor binding (Supplementary Figure S3E). As additional evidence, chromatin accessibility at the very same sites was also verified using publicly available ATAC-seq data from short-term melanoma cultures (Figure 3G, Supplementary Figure S3F–H; GSE134432). In line with this, promoter regions of genes associated with a Gain VEL showed increased levels of H3K27 acetylation and RNAPII occupancy in melanoma compared to NHEMs (Supplementary Figure S3I).

### 2.4. Genes Associated with AP-1-Bound Enhancers Are Involved in Melanoma Development

**Figure 4:**
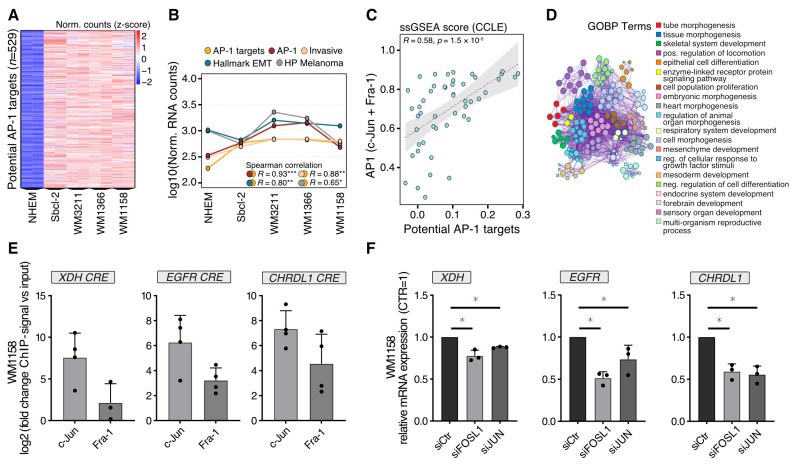
Functional influence of potential AP-1 targets on melanoma development (**A**) Heatmap illustrating normalized counts of potential target genes associated with an AP-1-bound Gain VEL and consistent upregulation in each melanoma cell line compared to NHEMs (*n* = 529). (**B**) Expression pattern comparison of the potential AP-1-regulated gene set (AP-1 targets) with AP-1 members c-Jun and Fra-1 (AP-1) and important gene sets associated with melanoma progression and invasiveness. (* *p* < 0.05, ** *p* < 0.01, *** *p* < 0.001). (**C**) Relationship between the potential AP-1 targets and AP-1 (c-Jun/Fra-1) in an additional 49 cutaneous melanoma cell lines obtained from the CCLE. Spearman’s R and *p*-value are indicated. (**D**) Functional gene set enrichment analysis of genes annotated to AP-1-bound Gain VELs (*n* = 529). (**E**) ChIP-qRT-PCR of the c-Jun/Fra-1-bound Gain VELs in WM1158 annotated to the potential target genes XDH, EGFR, and CHRDL1 (XDH CRE, EGFR CRE, CHRDL1 CRE) relative to input. (**F**) qRT-PCR after c-Jun- or Fra-1-siRNA knockdown in WM1158 shows significantly reduced expression of XDH, EGFR, and CHRDL1 (unpaired Student’s *t*-test, * *p* value < 0.05).

C-Jun was already shown to drive melanoma development and progression by direct regulation of genes involved in cancer-related signaling pathways [6]. To examine the role of genes putatively regulated by AP-1 binding to distant enhancers, we isolated genes assigned to a c-Jun/Fra-1-bound Gain VEL and observed significant upregulation in each of the four melanoma cell lines compared to NHEMs. Following this approach resulted in a set of 529 genes, showing consistently high expression in all melanoma cells (Figure 4A). Further, the expression pattern of the genes annotated to AP-1-bound Gain VELs not only showed a strong correlation with AP-1 members c-Jun and Fra-1 (AP-1; Spearman’s R: 0.93) but also with defined gene sets associated with melanoma progression and invasiveness (Invasive [25], Hallmark EMT and HP Melanoma (MSigDB, Version 2023); Figure 4B). Moreover, this strong relationship between c-Jun/Fra-1 and its potential target genes was supported by analysis of count data from an additional 49 cutaneous melanoma cell lines obtained from the CCLE (Figure 4C). Functionally, genes of the AP-1 target gene set were enriched in biological processes that are known to be of great importance in progressing melanoma cells, such as positive regulation of locomotion, morphogenesis, and negative regulation of cell differentiation (Figure 4D).

Next, we performed ChIP-qRT-PCR to experimentally confirm c-Jun/Fra-1 binding of Gain VELs. Therefore, we exemplarily selected 3 different c-Jun/Fra-1 bound Gain VELs annotated to the specific potential target genes *EGFR*, *XDH*, and *CHRDL1* without a c-Jun/Fra-1 ChIP-seq signal close to the TSS (±3 kb) to avoid regulatory influence by direct binding to the promoter. The qRT-PCR of the c-Jun or Fra-1 chipped samples (WM1158, WM1366) clearly showed AP-1 enrichment within the respective Gain VELs (XDH CRE, EGFR CRE, CHRDL1 CRE) relative to the input (Figure 4E, Supplementary Figure S4A, Table 1). Further, siRNA knockdown of either c-Jun or Fra-1 in WM1158 and WM1366 led to a significant decrease in *EGFR*, *XDH*, and *CHRDL1* expression, supporting a role for these AP-1 factors in regulating the selected genes (Figure 4F, Supplementary Figure S4B, Table 1, * *p* value < 0.05). Together with ChIP-qRT-PCR validation of c-Jun/Fra-1 occupancy, these data are consistent with the contribution of the identified distal regulatory regions to target gene expression by AP-1 binding. The respective siRNA knockdown efficiency was experimentally verified by qRT-PCR (Supplementary Figure S4C,D).

### 2.5. Expression of Genes Linked to AP-1-Bound Enhancer Regions Correlates with Poor Survival in BRAF Wild-Type Melanoma Patients

**Figure 5:**
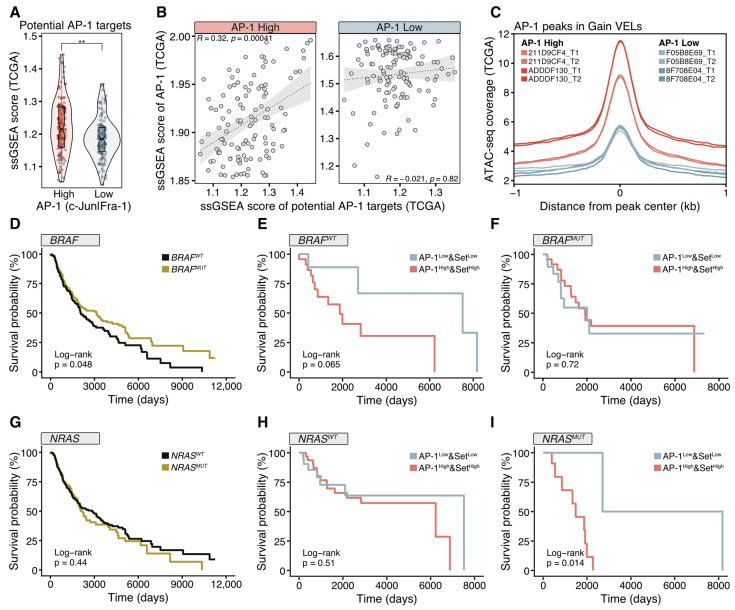
Clinical relevance of AP-1 and its potential targets in skin cutaneous melanoma (**A**) ssGSEA scores of potential AP-1 targets for melanoma patients (TCGA, Project ID: TCGA-SKCM) with high and low AP-1 (c-Jun/Fra-1) expression status. (** *p* < 0.01). (**B**) Relationship between the potential AP-1 targets and AP-1 (c-Jun/Fra-1) in AP-1^high^ and AP-1^low^ melanoma patients. Spearman’s R and *p*-value are indicated. (**C**) Histogram showing the ATAC-seq coverage of melanoma patients with AP-1^high^ and AP-1^low^ status at c-Jun/Fra-1 binding sites overlapping Gain VELs. (**D**) Overall survival analysis plot of melanoma patients based on *BRAF^WT^* and *BRAF^MUT^* status. (**E**,**F**) Overall survival analysis plot of *BRAF^WT^* (**E**) and *BRAF^MUT^* (**F**) melanoma patients based on the AP-1 expression level (AP-1) and enhancer-mediated AP-1 target gene set expression (Set). (**G**) Overall survival analysis plot of melanoma patients based on *NRAS^WT^* and *NRAS^MUT^* status. (**H**,**I**) Overall survival analysis plot of *NRAS^WT^* (**H**) and *NRAS^MUT^* (**I**) melanoma patients based on the AP-1 expression level (AP-1) and enhancer-mediated AP-1 target gene set expression (Set).

We next aimed to evaluate our data in a clinical context and thus analyzed skin cutaneous melanoma patient data (Project ID: TCGA-SKCM). Therefore, the patients were subdivided into AP-1^high^ and AP-1^low^ cohorts, followed by analysis of enhancer-mediated AP-1 target gene expression, clearly illustrating a significant increase in the AP-1^high^ cohort compared to AP-1^low^ (Figure 5A). Furthermore, a significant positive correlation between AP-1 and its potential targets was observed only in the AP-1^high^ group, while no correlation was detected in AP-1^low^ patients, supporting AP-1’s role in enhancing the expression of these potential targets (Figure 5B). Next, we examined TCGA ATAC-seq data for patients in each defined AP-1 cohort, showing significantly elevated chromatin accessibility at the identified c-Jun/Fra-1 binding sites within Gain VELs (Figure 5C).

Further, we were interested in the clinical impact of the AP-1-related gene-expression signature connected to melanoma-gained enhancer regions, focusing on patient cohorts stratified by mutation status, distinguishing between mutated and wild-type cases for the well-characterized mutations *BRAF* and *NRAS*. Interestingly, *BRAF^WT^* patients showed a lower survival probability than *BRAF^MUT^* patients, whereas patients classified according to *NRAS* illustrated the opposite trend (Figure 5D,G). Accordingly, survival analyses of either *BRAF^WT^* and *BRAF^MUT^* or *NRAS^WT^* and *NRAS^MUT^* based on the AP-1 expression level and expression of the putative AP-1-associated gene set clearly revealed a high impact on survival probability. The stratification indicated that high AP-1 and AP-1 target gene expression correlate with poorer clinical outcomes only in *BRAF* wild-type (*BRAF^WT^*/AP-1^high^ & Set^high^) patients (Figure 5E), with no significant effect observed in those harboring *BRAF* mutations (Figure 5F). For *NRAS*, however, the opposite was observed. Here, while mutations in the *NRAS* gene itself had only a minor influence on survival probability, high AP-1 and AP-1 target gene expression led to a significantly worse clinical outcome only in patients with *NRAS^MUT^* status (Figure 5H,I).

Collectively, these findings support our data gathered from cell line models and illustrate the clinical importance of high AP-1 expression levels in subsets of melanoma patients.

## 3. Discussion

The AP-1 family of transcription factors is known to be of great importance in melanoma development and progression, but a deeper mechanistic understanding and the identification of its target genes remain unsolved. In this study, we demonstrate that melanomagenesis is accompanied by profound epigenetic reprogramming, especially with regard to distal cis-regulatory elements. Notably, the loss of active regulatory elements was strongly associated with melanocytic differentiation, while the gain of active enhancers supported a dedifferentiated, invasive phenotype. Moreover, the integrative ChIP-seq and RNA-seq analyses show that AP-1, specifically c-Jun and Fra-1, preferentially binds melanoma-gained enhancers (Gain VELs) and associates with higher H3K27ac levels, RNAPII occupancy, and chromatin accessibility. These findings support an enhancer-centric model of AP-1 in malignant melanoma, extending prior work that implicated AP-1 in cancer biology through promoter-proximal regulation and signaling pathway output [23,26]. Clinically, high expression of the set of 529 genes annotated to c-Jun/Fra-1-bound Gain VELs correlated with poor overall survival in *BRAF^WT^* patients, emphasizing the potential of AP-1 as a diagnostic as well as therapeutic target in melanoma treatment.

Motif-centered analyses indicate that ~80% of the c-Jun/Fra-1 peaks in Gain VELs harbor a central AP-1 motif, typically one motif per peak, arguing for sequence-specific binding and regulation by discrete, high-affinity dimers rather than the formation of large, multimeric complexes. These findings, along with the strong bimodal H3K27ac distribution and open chromatin around c-Jun/Fra-1 binding sites, suggest a mechanism in which AP-1 establishes or maintains active enhancers in a pioneer-factor-like manner. AP-1 is known to co-occupy enhancers with histone acetyltransferases and to take a critical role in chromatin accessibility maintenance at regulatory elements [27,28,29,30]. In other solid tumors, such as breast and colorectal cancer, members of this family, particularly Fra-1, have already been shown to establish super-enhancers supporting oncogenic transcription, and that overexpression of Fra-1 drives super-enhancer activation, as evidenced by increased H3K27ac levels [31,32]. Connected to this, our data also revealed significantly higher H3K27ac levels at Gain VELs bound by c-Jun/Fra-1 in melanoma compared to NHEMs, further pointing towards the biologically meaningful importance of these enhancers. Moreover, Fra-1 was found to be required for efficient RNAPII recruitment at the *HMGA1* promoter in triple-negative breast cancer [33]. In line with this, the achieved results illustrate that promoters of genes associated with AP-1-bound Gain VELs showed a considerable increase in RNAPII and H3K27ac ChIP-seq signals. In this regard, our data newly hint at a comparable process in melanoma, in which c-Jun and Fra-1 do not function independently but likely recruit chromatin remodelers, such as p300 or CBP, to open chromatin at melanoma-specific enhancer sites and support RNAPII recruitment at respective gene promoters. Beyond this, the high degree of co-occupancy between c-Jun and Fra-1 strongly argues that these factors predominantly bind as c-Jun/Fra-1 heterodimers at distal regulatory elements. While c-Jun is able to form homodimers, Fra-1 lacks a transactivation domain and depends on partners like c-Jun to regulate gene expression. This implies a dependency, where c-Jun drives transcriptional regulation while Fra-1 increases c-Jun’s activity by reinforcing complex stability. This assumption is supported by recent studies, in which Fra-1 has been shown to stabilize c-Jun in response to RAS/MAPK signaling by inhibiting its proteasomal degradation [34]. The resulting stabilization enhances the accumulation and chromatin binding of c-Jun/Fra-1 complexes, consistent with our findings of high occupancy and concurrent enrichment of H3K27ac and RNAPII at Gain VELs. Additionally, knockdown of c-Jun or Fra-1 reduced the expression of selected genes annotated to c-Jun/Fra-1-bound Gain VELs, strengthening the link between AP-1 activity and expression of these genes. Still, these knockdowns cannot distinguish direct effects from indirect downstream consequences of AP-1 depletion, nor prove that the assigned distal CREs mediate the observed expression changes. Direct enhancer-specific perturbation would be required to establish whether individual AP-1-bound distal elements are necessary for target expression.

At the level of gene networks, AP-1-bound Gain VELs are associated with a coherent program of genes involved in a pro-tumorigenic module, providing a link between oncogenic signaling and phenotypic plasticity in melanoma. Potential target genes are connected to biological processes like cell migration, morphogenesis, and extracellular matrix remodeling, and show expression patterns consistent with invasive transcriptional states. This observation is concordant with prior work placing AP-1 at the center of transcriptional programs that promote plasticity and metastasis in melanoma and other cancers [14,35]. Notably, motif analysis of all Gain VELs also revealed enrichment of TEAD and ETS motifs, suggesting recurrent involvement of these transcription factor families to refine target selection and functional output. Such cooperation seems biologically plausible as several studies point to complementary rather than redundant roles of TEAD and ETS in melanoma [25,36,37,38,39,40]. Thus, even when analyses were restricted to AP-1-bound Gain VELs, the presence of ETS/TEAD motifs argues for a combinatorial mode in which AP-1 plays a central activating role. Furthermore, the genes we validated experimentally illustrate that selected members of the AP-1-associated gene set are responsive to c-Jun/Fra-1 depletion and are linked to AP-1-bound distal regulatory regions. *EGFR* is a well-characterized driver of aggressive, dedifferentiated melanoma phenotypes and therapeutic resistance, as increased levels have been correlated with acquired BRAFi/MEKi resistance and enhanced migration [41,42]. *XDH* mRNA levels could recently be correlated with the prognosis of several human cancers. In HCC, the downregulation of *XDH* was an independent survival predictor associated with worse prognosis [43,44]. However, in agreement with our results, *XDH* was highly expressed in lung adenocarcinoma and was significantly correlated with poor clinical outcome [43,44]. The BMP antagonist *CHRDL1* was also proven to be involved in tumor biology by regulating EMT and metastasis. In contrast to other studies, in which low *CHRDL1* levels contribute to enhanced migration and poor survival, we observed significantly upregulated expression in melanoma cells [45,46]. Together, these gene validations give functional plausibility to the derived biological processes and support the idea that AP-1 coordinates a transcriptional program that promotes tumor development via melanoma-gained enhancers.

In clinical cohorts (TCGA-SKCM), a high AP-1 status is associated with markedly increased expression and demonstrates a positive correlation with its putative target gene set, while simultaneously showing enhanced chromatin accessibility at c-Jun/Fra-1 binding sites. Collectively, these findings demonstrate that the target genes identified in vitro are also related to high AP-1 activity and increased chromatin accessibility at AP-1-bound regulatory regions in patient samples. Although high AP-1 expression and high expression of the related gene set correlated with poorer survival in defined genetic subgroups, these analyses do not establish that AP-1 activity is an independent prognostic factor. Potential confounders such as tumor stage, metastatic site, treatment history, mutational burden, and other clinical variables may contribute to the observed survival differences. However, recent studies revealed that the MAPK/ERK signaling pathway is constitutively activated and tightly coupled to AP-1 activity in *BRAF^MUT^* melanomas [47,48]. For c-Jun, it has been shown that enhanced signaling causes activation via phosphorylation and that increased c-Jun abundance correlates with metastatic potential and BRAF inhibitor resistance [49,50]. Our observation, however, suggests that in the absence of *BRAF^MUT^*-caused activated MAPK signaling, tumors may rely more heavily on an AP-1-regulated gene expression network linked to distal regulatory elements to sustain invasive characteristics. This classifies AP-1 not only as a potential biomarker to stratify *BRAF^WT^* patients but as a potential therapeutic target. Strategies inhibiting AP-1 directly could be particularly effective in *BRAF^WT^* patient cohorts in which this pathway correlates with poorer clinical outcomes.

Several limitations of this study should be considered. First, it should be noted that comparisons between cultured NHEMs and melanoma cell lines may be influenced not only by malignant transformation but also by differences in differentiation state, lineage context, and culture conditions. Furthermore, melanin content and melanogenic activity were not directly quantified and may represent an additional source of biological variation, given their context-dependent effects on redox homeostasis, cellular metabolism, melanoma behavior and treatment response [51]. However, the four melanoma cell lines utilized within this study were maintained under comparable conditions, and differences in their origin, tumor stage, and genetic background were deliberately included to identify AP-1-associated regulatory features that are recurrent across heterogeneous melanoma models rather than restricted to a single cellular context. Therefore, the observation that c-Jun/Fra-1-associated Gain VEL patterns and the linked gene expression program were detected across these distinct melanoma cell lines supports the robustness of the identified AP-1-associated regulatory signature. Secondly, enhancer–gene relationships were inferred using only genomic annotation and proximity-based approaches; hence, the assigned genes should be considered putative AP-1-associated genes rather than experimentally validated enhancer targets. A limitation of such annotation-based approaches is that distal enhancers may regulate genes that are not in proximity or that are in contact with multiple promoters, leading to incomplete or inaccurate enhancer–gene assignments. This requires further experiments such as chromosome conformation capture or reporter assays to directly link enhancers with their target promoters.

In conclusion, our findings position AP-1 as an important contributor to malignancy and melanoma development by mediating a pro-invasive transcriptional program via melanoma-gained enhancers. This enhancer-mediated dependency is particularly pronounced in *BRAF^WT^* tumors, where high AP-1 activity and expression of the associated gene set correlate with poorer clinical outcomes. While additional functional and chromatin-contact studies are required to establish direct enhancer-specific causality, our data provide a framework for understanding AP-1-associated regulatory remodeling in melanoma as well as for identifying candidate transcriptional dependencies and novel therapeutic targets beyond the canonical MAPK/ERK pathway.

## 4. Materials and Methods

### 4.1. Cell Culture

Human melanoma cell lines Sbcl-2 (RRID: CVCL_D732), WM3211 (RRID: CVCL_6797), WM1366 (RRID: CVCL_6789), and WM1158 (RRID: CVCL_6785) (a generous gift from Dr. M. Herlyn, Wistar Institute, Philadelphia, PA, USA) were maintained in a culture medium consisting of MCDB153 (Sigma-Aldrich, Steinheim, Germany) with 20% Leibovitz’s L-15 (PAA Laboratories, Coelbe, Germany), 2% FCS, 1.68 mM CaCl2 (Sigma), and 5 µg/mL insulin (Sigma-Aldrich) at 37 °C and 5% CO_2_. NHEMs were derived from neonatal foreskin (NHEMs, PromoCell, Heidelberg, Germany, or Thermo Fisher Scientific, Waltham, MA, USA) and were cultured in melanocyte growth media as defined by the supplier at 37 °C and 5% CO_2_. Cell line characteristics concerning cell stage and mutation status were described previously [12].

### 4.2. Cell Transfection

The melanoma cell lines were transfected with an siRNA pool against JUN or FOSL1 (siTools Biotech GmbH, Planegg, Germany) and a siCtrl for 48 h, respectively, using the Lipofectamine RNAiMAX reagent (Life Technologies, Darmstadt, Germany). SiRNA pools consist of multiple siRNAs, resulting in efficient target gene knockdown with minimal off-target effects.

### 4.3. qRT-PCR

For gene expression analysis, RNA from melanoma cell lines and NHEMs was extracted using the EZNA Total RNA Kit I (Omega Bio-Tek, Inc., Norcross, GA, USA) before generating cDNA via reverse transcription using SuperScript II Reverse Transcriptase (Invitrogen, Groningen, The Netherlands). qRT-PCR was performed on a LightCycler (Roche, Mannheim, Germany) as described previously [6]. ß-Actin was used for normalization.

For ChIP qRT-PCR, Input DNA as well as c-Jun and Fra-1 immunoprecipitated DNA were used. Primer efficiency (PE) was determined for the respective primer sets (PE_CHRDL1_CRE_: 2.015; PE_EGFR_CRE_: 1.951; PE_XDH_CRE_: 1.959). ChIP enrichment was verified using quantitative qRT-PCR. Enrichment values were normalized to the respective input DNA and expressed as log2 fold change. ChIP-negative primers (NegCTR) served as controls, and here, too, c-Jun/Fra-1 enrichment values were normalized to the respective input DNA and reported as mean log2 fold-change (NegCTR_cJun_WM1366_: −5.16; NegCTR_Fra1_WM1366_: −1.54; NegCTR_cJun_WM1158_: −3.51; NegCTR_Fra1_WM1158_: −4.14; *n* = 3). Product length was controlled on an agarose gel, and the correct amplification region was confirmed by sequencing.

Annealing and melting temperatures were optimized for each primer set (Table 1).

### 4.4. RNA-Seq Analysis

RNA-seq data used for downstream analyses were generated by our working group and previously published in the Gene Expression Omnibus (GEO) under accession number GSE284779. For each cell line, two biological replicates were sequenced. Read quality was assessed utilizing FastQC (v0.12.1; SCR_014583) before performing adapter trimming and quality filtering with fastp (v0.23.4) [52]. Reads were mapped against the reference genome (hg38) using the STAR (v2.7.11a; SCR_004463) aligner and GENCODE human transcript annotation (v44) [53]. Sorted BAM files were indexed with samtools (v1.17) index, and mapping statistics were obtained via samtools flagstat [54]. An RNA-based count matrix was generated using featureCounts (v2.0.6; SCR_012919) before running a differential gene expression analysis following the DESeq2 (v1.48.2) pipeline in R (v4.5.1; SCR_001905) [55,56]. Genes with an FDR < 0.1 and an absolute log2 fold change > 0 were further considered as differentially expressed. Counts per million (CPM) normalized bigWig files were generated using deepTools (v3.5.3; SCR_016366) bamCoverage [57]. The z-score-scaled gene expression heatmap was created using the R package pheatmap (v1.0.13) on rlog-transformed (DESeq2) count data. Statistically significant enriched Gene Ontology Biological Process (GOBP) terms were identified using Metascape (v3.5; SCR_016620), and the resulting interaction network was visualized in Cytoscape (v3.10.4; SCR_003032) for clarity [58,59]. The statistical significance of GO enrichment was evaluated using a hypergeometric test, and *p*-values were corrected for multiple testing using the Benjamini–Hochberg method. Other downstream explorative analyses were performed in R, and all remaining plots were generated with ggplot2 (v4.0.0; SCR_014601) [60].

### 4.5. ChIP-Sequencing

Chromatin immunoprecipitation (ChIP) was carried out as previously described [12]. ChIP was performed using antibodies specific to H3K27ac (AB_2118291, Abcam, Cambridge, UK) and RNAPII (AB_2732926, Active Motif, Carlsbad, CA, USA). ChIP-seq libraries were generated using the TruSeq ChIP Library Preparation Kit (IP-202-1012, Illumina Inc., San Diego, CA, USA) following the recommended workflow. Library quality and fragment size distribution were assessed using a 4200 Tape Station (Agilent Technologies, Santa Clara, CA, USA), and concentrations were determined by Qubit fluorometric quantification. Prepared libraries were sequenced on an Illumina NextSeq2000 using a P4 Flow-cell with 50 bp single-end reads. H3K27ac ChIP-seq experiments were performed in biological triplicates. Transcription factor ChIP-seq was conducted with c-Jun or Fra-1 antibodies in the four melanoma cell lines, which served as biological replicates.

### 4.6. ChIP-Sequencing Analysis

For downstream analyses, we used both newly generated and previously published ChIP-seq datasets (PRJNA1197724) produced by our working group. In total, three biological replicates were generated for H3K27ac per cell line, of which two samples (n1 and n2) were newly prepared, and one sample (n3) was reanalyzed. Two biological replicates were newly created for RNAPII per cell line. C-Jun and Fra-1 ChIP-seq data of each melanoma cell line utilized within this study were reanalyzed. After the initial quality check of all ChIP-seq data, single-end ChIP-seq reads were aligned to the human reference genome (hg38, v44) using bowtie2 (v2.5.1; SCR_016368) and reads with a mapping quality lower than 10 were removed using samtools view [61]. SAM files were converted into BAM files with samtools and reads aligning to highly repetitive regions according to the ENCODE blacklist (hg38-blacklist.v2) were excluded with bedtools intersect (v2.30.0; SCR_006646) [62]. Replicate correlation between H3K27ac ChIP-seq samples was analyzed using deepTools functions multiBigwigSummary and plotCorrelation. 1× normalized (RPGC) bigWig tracks were created utilizing deepTools bamCoverage, and ChIP-seq heatmaps were generated with computeMatrix and plotHeatmap. BigWig files from biological replicates were merged using wiggletools mean function. Peak calling was performed using HOMER’s (v4.10; SCR_010881) findPeaks program in “factor” mode for transcription factors, with FDRs of 10^−3^ and 10^−6^ for c-Jun and Fra-1, respectively [63]. H3K27ac ChIP-seq peaks were called using HOMER’s getDifferentialPeaksReplicates.pl program, including all biological replicates per cell line. For all newly generated ChIP-seq datasets, quality control metrics were assessed, including overall alignment rate and uniquely mapped reads, duplication rate, IP efficiency defined as the fraction of tags within peaks relative to genomic background, and normalized strand cross-correlation coefficient (NSC). A summary of these metrics is provided in Supplementary Table S1. Overlapping peaks were identified with HOMER’s mergePeaks program. ChIP-seq peak annotation to the nearest transcription start site was done using ChIPseeker (v1.44.0) in default mode with the basic GENCODE annotation (v44) file filtered for protein-coding genes. GREAT (v4.0.4; SCR_005807) was used in default mode to annotate H3K27ac ChIP peaks to potential target genes [64]. Potential enhancer-based PCA was done using the prcomp function in R. RNAPII occupancy at Gain and Loss VELs for NHEMs and melanoma cell lines was analyzed using deepTools computeMatrix and plotProfile functions. To evaluate c-Jun/Fra-1 binding relative to the H3K27ac signal in Gain VELs, tag directories from all melanoma cell lines were combined for c-Jun, Fra-1, and H3K27ac using the makeTagDirectory program from HOMER, followed by read coverage calculation with HOMER’s annotatePeaks.pl and scaling in R by dividing each value by the observed maximum.

### 4.7. Differential Enrichment Analysis

To identify differentially enriched cis-regulatory regions, i.e., variant enhancer loci (VELs), between melanocytes and melanoma cells, the H3K27ac peaks for each cancer cell line were first merged with the NHEM H3K27ac peaks to generate cell line-specific consensus peak sets. The DESeq2 R package was then used to identify regions with a statistically significant enrichment of H3K27ac between the individual melanoma cell lines and NHEM, with three replicates available for each condition. Accordingly, the counts were normalized using DESeq2’s median-of-ratios method, and the dispersion estimates were determined using a parametric mean–variance model with empirical Bayesian shrinkage. VELs were defined as regions with an FDR < 0.05 and an absolute log2 fold change > 1.

### 4.8. Motif Analysis

Motif discovery to determine transcription factors potentially involved in *VEL* regulation was done by centering H3K27ac peaks on the nucleosome-free region (NFR) with the getPeakTags from HOMER. Subsequently, de novo motif analysis was conducted using HOMER’s findMotifsGenome.pl function within a 300 bp window around the NFR center. For overlapping c-Jun/Fra-1 peaks in Gain VELs, a de novo motif search was performed with findMotifsGenome.pl using a 200 bp peak-centered region. The de novo AP-1 motif density within c-Jun/Fra-1 peaks was analyzed using the -hist 1 -m options of HOMER’s annotatePeaks.pl program and visualized in R using ggplot2. The number of AP-1 motifs found per c-Jun/Fra-1 peak in a 200 bp window was determined utilizing the -nmotifs option of the same program.

### 4.9. Bioinformatical Integration of External Data Sets

BigWig files for both the H3K4me1/me3 ChIP-seq data from SKmel147 and NHM (GSE94488), as well as the ATAC-seq data from melanoma cultures MM029 and MM099 (GSE134432), were obtained from the GEO database under the specified accession number. The files were moved from the hg19 to the hg38 assembly with USCS LiftOver (v482) [65]. All corresponding heatmaps were generated with deepTools. Bed files for Candidate Promoter-like (PLS) and Candidate Enhancers (ELS) cis-Regulatory Elements (cCREs) by ENCODE were downloaded from the SCREEN webpage (https://screen.wenglab.org accessed on 13 October 2025) [66]. Overlap between these cCREs and potential enhancers was analyzed with HOMER’s mergePeaks. The hg38 lifted 15-state core chromatin model of melanocytes (E061) was retrieved from the NIH Roadmap Epigenomics Mapping Consortium [67], and the total genomic chromatin state was compared with the chromatin state at sites overlapping with potential enhancers identified in NHEMs using a custom R script. Giggle scores, indicating the similarity of overlaps between factors deposited in the Cistrome Data Browser (DB) and the Gain and Loss VELs [68], were determined with the Cistrome DB Toolkit (http://dbtoolkit.cistrome.org accessed on 23 October 2025). Regulatory activity modeling was performed using the webtool CREMA (https://crema.unibas.ch/crumara/ accessed on 2 July 2026) on raw H3K27ac ChIP-seq signal profiles from NHEMs and the four melanoma cell lines. Samples were subsequently aggregated into NHEM and melanoma groups using the CREMA averaging functionality. CREMA-inferred activity scores were extracted from the generated activity tables. Differential regulatory activity was assessed by comparing melanoma and NHEM activity scores for active regulatory matrices. The Cancer Cell Line Encyclopedia (CCLE) metadata and RPKM-normalized gene expression data were obtained from the Broad Institute (https://sites.broadinstitute.org/ccle/ accessed on 26 April 2024). Single-sample GSEA (ssGESEA) scores for the AP-1-regulated gene set were determined for all CCLE melanoma cell lines using the GSVA (v2.2.1; SCR_021058) package in R [69], and correlation with AP-1 expression was calculated with ggpubr (v0.6.1; SCR_021139). Transcript-per-million (TPM) normalized count data of the TCGA SKCM were obtained from GDC using the TCGAbiolinks (v2.36.0; SCR_017683) function in R [70,71]. SsGSEA scores for the AP-1-regulated gene set and AP-1 (c-Jun and Fra-1) were determined, and correlation was calculated as above. Normalized ATAC-seq tracks for available TCGA SKCM patients were downloaded from the GDC database [72] and visualized with deepTools. Survival analyses were performed using the survival package in R. Patients were categorized as “high” and “low” if the ssGSEA score for AP-1 or the AP-1 gene set expression was equal to or higher than the 0.75 quantile or lower than or equal to the 0.25 quantile, respectively.

### 4.10. Statistical Analysis

Statistical analysis of experimental results was executed using GraphPad Prism 10 software (Version 10.1.1; GraphPad Software Inc., San Diego, CA, USA). If not stated otherwise, all results are normalized to the respective control and shown as mean ± SEM (range). Comparison between the groups was made by using Student’s unpaired *t*-test. All experiments were repeated at least three times in an independent manner. A critical *p*-value of *p* < 0.05 was considered statistically significant if not stated otherwise. No indication between groups implies no statistical significance.

## Figures and Tables

**Table 1:** Oligonucleotides used for real-time PCR.

Gene	Forward Primer	Reverse Primer
CHRDL1	GGCAACCCAATCAATGCACC	CTTGCTTCTCTGTTGGCAGG
EGFR	GCGCCTTGACTGAGGACAGCATAGACGA	GGAGCCCTTAAAGATGCCATTTGGCTTGGC
XDH	TCTATGCGGCTTGTCAGACC	CAAGCCACCCCATAGCTGAA
CHRDL1 CRE	AGCAAACCTGACAGTCCTCA	GACTCTCCAGGCAGTGAATCAT
EGFR CRE	TCGATGACTCTGCTTGGTGC	GTGTTGACGCAGAGCTGGAT
XDH CRE	TGCAGGGCTGTTCCTTAGC	TCATGTTGCCTGGGGGTTTC
NegCTR	TTTCGTTTTGCCCAGGTCCAT	AAGGAGTAGGTGTGACTTCCAT

## Data Availability

The RNA- and ChIP-sequencing data generated and/or analyzed during the current study are publicly available in the NCBI Gene Expression Omnibus (GEO) repository under accession numbers GSE284779 and GSE326324. All relevant data supporting the findings of this study can be accessed without restriction through this repository. Additional processed data and information required to reproduce the analyses may be requested from the corresponding authors upon reasonable request.

## Supplementary Materials

The following supporting information can be downloaded at: https://www.mdpi.com/article/10.3390/ijms27146459/s1.
