# Supplementary material for: Transcriptional Reprogramming by AP-1-Bound Cis-Regulatory Elements Is Associated with Melanoma Development
Source: Int J Mol Sci. 2026 Jul 21;27(14):6459. doi: 10.3390/ijms27146459 (PMC13411661; doi:10.3390/ijms27146459)
Supplement: Supplementary file 1 [file ijms-27-06459-s001.zip › Supplementary_Information.pdf]

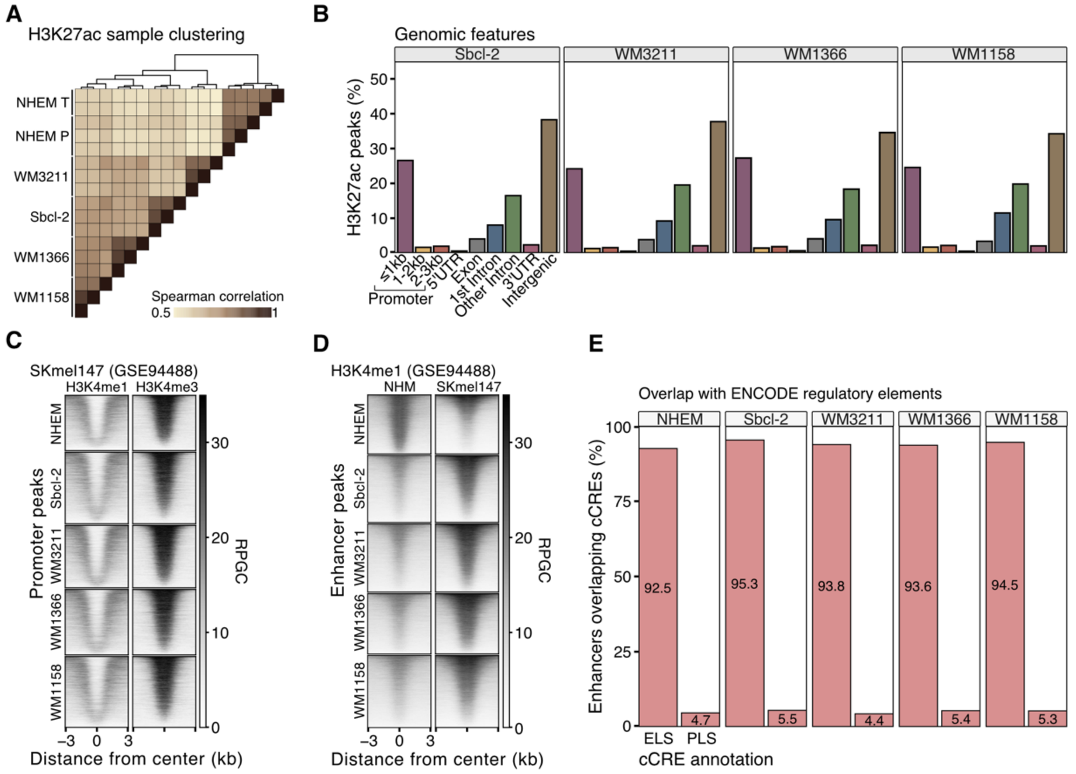

Supplementary Figure 1: Verification of identified enhancers in NHEMs and melanoma cells. **(A)** Correlation-based unsupervised clustering of genome-wide H3K27ac ChIP-seq samples, each represented by biological triplicates. **(B)** Distribution of Sbcl-2, WM3211, WM1366, and WM1158-derived H3K27ac ChIP-seq peaks in annotated genomic features. **(C)** Histogram showing the H3K4me1 and H3K4me3 ChIP-seq signal of publicly available data from the melanoma cell line (SKmel147) (GSE94488) around the defined promoter peaks in NHEMs, Sbcl-2, WM3211, WM1366, and WM1158. **(D)** Histogram showing the H3K4me1 ChIP-seq signal of publicly available data from the melanoma cell line (SKmel147) and melanocytes (NHM) (GSE94488) around the defined enhancer peaks in NHEMs, Sbcl-2, WM3211, WM1366, and WM1158. **(E)** Overlap of ENCODE enhancer (ELS) and promoter (PLS) annotations (cCREs) from all available bio samples with potential enhancer elements.

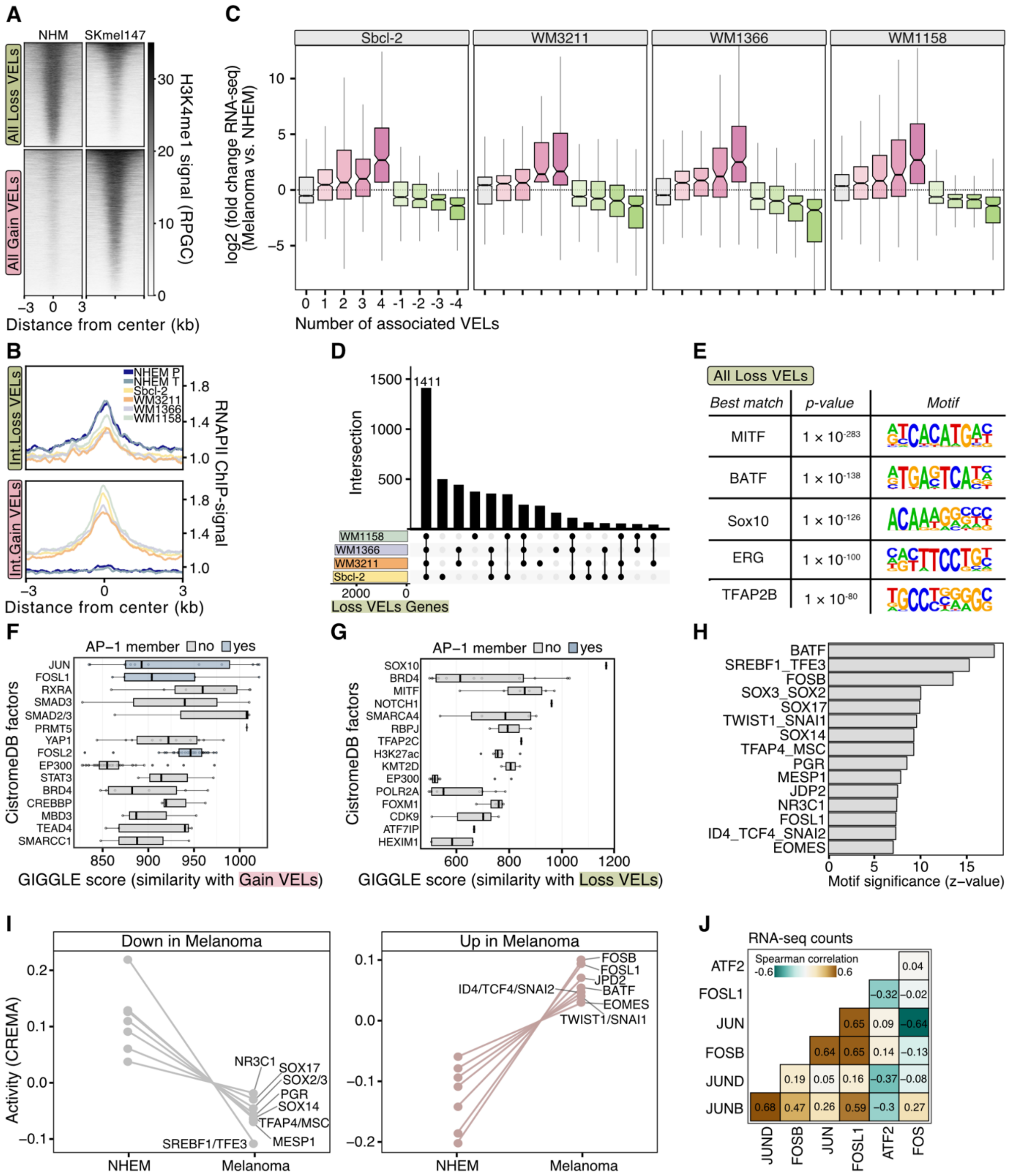

Supplementary Figure 2: **Characteristics of Gain and Loss VELs in melanoma. (A)** Histogram showing the H3K4me1 ChIP-seq signal of publicly available data from the melanoma cell line (SKmel147) and melanocytes (NHM) (GSE94488) around all Loss and Gain VELs. **(B)** RNAPII ChIP-seq signal intensity around the enhancer center in intergenic (int.) Loss and Gain VELs in NHEMs (NHEM_P, NHEM_T) and melanoma cell lines. The signals for the two biological RNAPII ChIP-seq samples per cell line were merged. **(C)** Log2 fold-change RNA-seq expression values of genes assigned to No VEL, and different numbers of associated Loss VELs and Gain

VELs. **(D)** Intersection of annotated target genes to Loss VELs in melanoma cell lines. Number of genes found in all cell lines is indicated. Total gene numbers are shown in horizontal bar graphs. **(E)** Motifs derived from de novo motif analysis enriched in all Loss VELs. P-values and transcription factors showing the best match with the de novo motifs are indicated. **(F)** Similarity between binding sites of factors deposited in CistromeDB and all Gain VELs is depicted as GIGGLE score. **(G)** Similarity between binding sites of factors deposited in CistromeDB and all Loss VELs, depicted as GIGGLE score. **(H)** Bar plot depicting the top 15 regulatory motifs identified by CREMA, sorted by significance (z-value). The z-value summarizes the importance of the motif explaining the observed signal intensity variation across the samples. **(I)** CREMA activity profiles for the regulatory motifs shown in H, grouped according to activity decrease and increase in melanoma samples compared to NHEMs, respectively. **(J)** Correlation between AP-1 family members based on normalized RNA-seq counts.

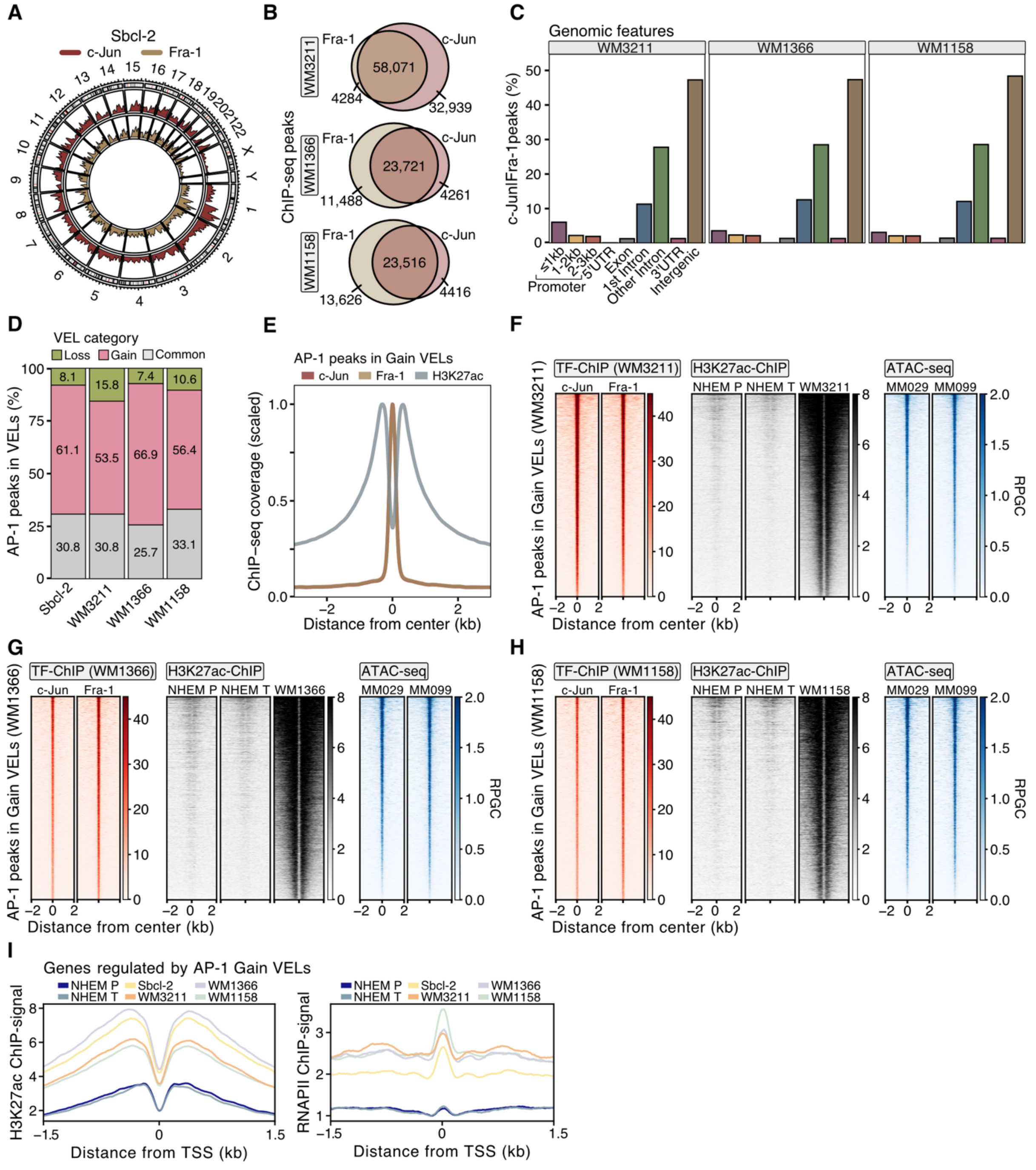

Supplementary Figure 3: **c-Jun/Fra-1 binding sites overlapping Gain VELs. (A)** Circos Plot illustrating the genomic distribution of c-Jun and Fra-1 binding sites in Sbcl-2. **(B)** Venn Diagram showing the overlap between c-Jun and Fra-1 binding sites in WM3211, WM1366, and WM1158 (PRJNA1197724). **(C)** Distribution of WM3211, WM1366, and WM1158-derived c-Jun|Fra-1 ChIP-Seq peaks in annotated genomic features. **(D)** Proportion of c-Jun/Fra-1 peaks overlapping with Loss VELs, Gain VELs, and Common VELs in individual melanoma cell lines. **(E)** Histogram showing the H3K27ac ChIP-seq and c-Jun/Fra-1 ChIP-seq signal around c-

Jun/Fra-1 binding sites overlapping Gain VELs. ChIP-seq coverage was rescaled for visualization purposes. **(F-H)** Histograms showing the signals of c-Jun/Fra-1 ChIP-seq, H3K27ac ChIP-seq, and ATAC-seq (GSE134432) at c-Jun/Fra-1 binding sites overlapping Gain VELs in WM3211 **(F)**, WM1366 **(G)**, and WM1158 **(H)**. **(I)** Histograms showing the signals of H3K27ac ChIP-seq and RNAPII ChIP-seq around the TSS of genes potentially regulated by AP-1-bound Gain VELs in NHEMs and melanoma cell lines.

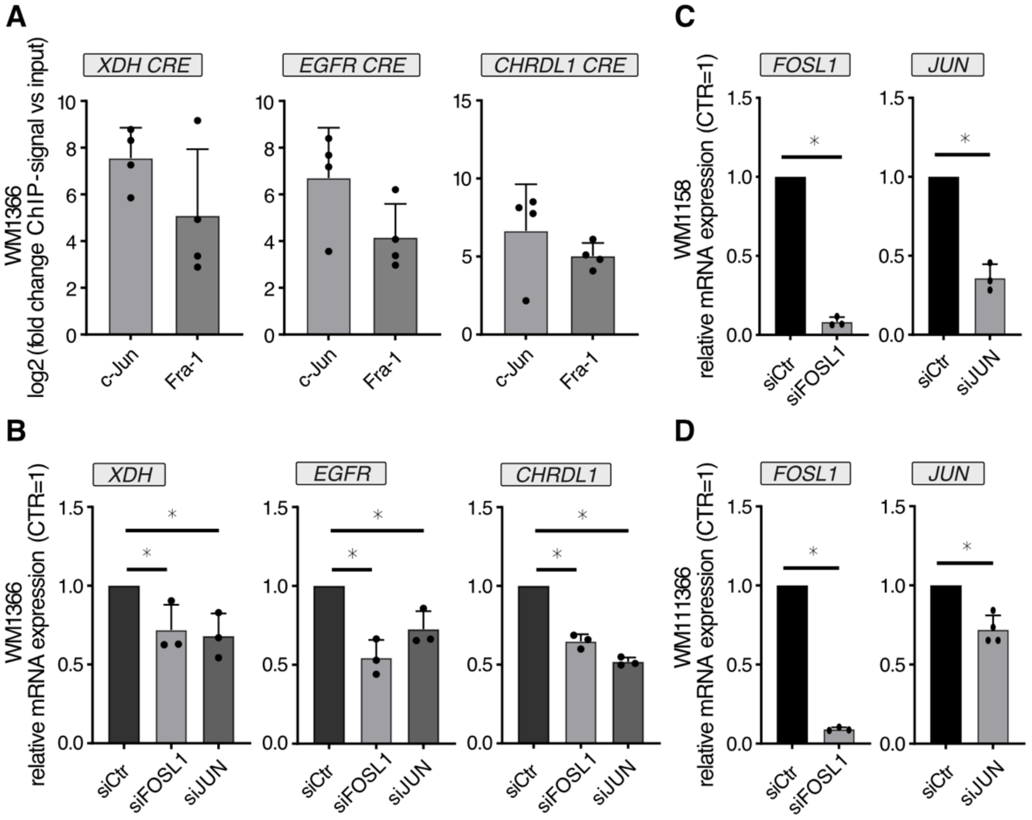

Supplementary Figure 4: Validation of c-Jun/Fra-1 occupancy at selected Gain VELs and regulation of associated genes. (A) ChIP-qRT-PCR of the c-Jun/Fra-1-bound Gain VELs in WM1366, annotated to the potential target genes *XDH*, *EGFR*, and *CHRDL1* (XDH CRE, EGFR CRE, CHRDL1 CRE relative to input (log2FC). **(B)** qRT-PCR after c-Jun- or Fra-1- siRNA knockdown in WM1366 shows significantly reduced expression of *XDH*, *EGFR*, and *CHRDL1* gene expression (unpaired students t-test, *pval<0.05). Knockdown efficiency in WM1158 **(C)** and WM1366 **(D)** determined by qRT-PCR after c-Jun or Fra-1 siRNA transfection (unpaired Student's t-test, *pval<0.05).
